# Impact of Effectiveness of Physical Activity in a Virtual Environment on the Regulation of Sclerostin and Interleukin 6 Levels in Haemodialysis Patients

**DOI:** 10.3390/jcm13082321

**Published:** 2024-04-17

**Authors:** Agnieszka Turoń-Skrzypińska, Alicja Mińko, Aleksandra Rył, Katarzyna Mańkowska, Kazimierz Ciechanowski, Zuzanna Bereda, Iwona Rotter, Grażyna Dutkiewicz

**Affiliations:** 1 Department of Medical Rehabilitation and Clinical Physiotherapy, Pomeranian Medical University, 71-210 Szczecin, Poland; agi.skrzypinska@gmail.com (A.T.-S.); aleksadra.ryl@pum.edu.pl (A.R.); zuzanna.bereda@pum.edu.pl (Z.B.); iwrot@wp.pl (I.R.); 2 Department of Microbiology, Immunology and Laboratory Medicine, Pomeranian Medical University, 70-111 Szczecin, Poland; katarzyna.mankowska@pum.edu.pl; 3 Department of Nephrology, Transplantology and Internal Medicine, Pomeranian Medical University, 70-111 Szczecin, Poland; kazimierz.ciechanowski@pum.edu.pl (K.C.); grazyna.dutkiewicz@pum.edu.pl (G.D.)

**Keywords:** hemodialysis, interleukin, sclerostin, virtual reality

## Abstract

**Background:** Chronic kidney disease is a significant public health issue associated with reduced physical activity. This can lead to mineral and bone disorders and increased levels of inflammatory markers. One innovative solution that can significantly contribute to increasing patient motivation is the combination of physical training with virtual reality technology during haemodialysis sessions. The aim of this study is to comprehensively assess the impact of regular virtual reality-based physical activity on plasma sclerostin and interleukin 6 levels, as well as on physical performance and the level of physical activity in patients undergoing renal replacement therapy through haemodialysis. **Methods:** This study is a prospective cohort study. Patients included in the study were randomly assigned to two groups: the study group and the control group. The study group consisted of patients who were entrusted with the task of conducting training using the prototype of the NefroVR system. The duration of the study period for both the study and control groups was 3 months. **Results:** One hundred and two (102) patients with stage 5 chronic kidney disease who underwent haemodialysis as a renal replacement therapy participated in the study. Patients from the study group were characterized by higher physical activity compared to the control group. There was a significant difference in the level of IL-6 and SOST between the study and control groups in the second measurement. **Conclusions:** Regular physical activity, especially using approaches such as virtual reality, contributes to improving physical fitness and overall activity levels in patients undergoing haemodialysis. The study demonstrated that regular exercise may be associated with a reduction in inflammatory parameters and positive effects on bone metabolism in patients undergoing haemodialysis.

## 1. Introduction

Chronic kidney disease (CKD) is a significant public health issue associated with reduced physical activity [1,2,3,4]. This can lead to mineral and bone disorders and increased levels of inflammatory markers [1].

Sclerostin, which is a glycoprotein encoded by the 17q12-q21 region of chromosome 17 near the *SOST* gene, plays a vital role in regulating bone mass and limiting anabolic processes associated with bone formation [2,3,4]. Mechanical load is a key regulator controlling bone formation and remodelling involving osteocytes. Its elevated concentrations is observed, above all, in patients with end-stage renal failure, contributing to bone remodelling disorders. Studies have demonstrated that its levels in that group of patients is several times higher than that in a population with normal renal function. Regular physical activity stimulates osteogenesis and myogenesis, whereas inactivity prompts adipogenesis. The bone- and muscle-building effects are the result of the muscle pump mechanism (muscle contraction and relaxation) that inhibits the synthesis of sclerostin. Muscle mass increases and decreases in parallel with bone mineral density (BMD). Osteopenia is associated with sarcopenia [5,6,7,8,9].

The rise in interleukin 6 (IL-6) levels, a pleiotropic cytokine encoded by the short arm of chromosome 7 (7p15-p21), is associated with inflammation and reduced muscle clearance. Inflammations may lead to as high as a 100-fold increase in IL-6 concentration. IL-6 is mostly cleared by the kidneys and the liver [4,10]. The increase in IL-6 levels is particularly evident in patients undergoing haemodialysis. This inflammatory state may result from chronic kidney disease, cardiovascular diseases, or complications arising from haemodialysis treatment. Chronic kidney disease patients are at an increased risk of infections, the ethology of which may be related to bacterial and viral infection prevalence. The presence of various foreign bodies, including the necessary dialysis catheters and arteriovenous fistulas, integral to renal failure treatment, contributes to inflammatory reactions in the population. The dialysis process itself may also elevate the risk of infection, especially with improper hygiene or sterilization procedures. Additionally, patients with endstage renal failure demonstrate increased levels of pro-inflammatory cytokines, partly due to protein deficiencies common in this group. Reduced muscle clearance typical for CKD leads to the accumulation of these substances in the body, which might further intensify the inflammatory response [11,12,13].

Deteriorated health, reduced well-being, and the necessity to undergo renal replacement therapy, i.e., haemodialysis, often result in difficulties for patients in engaging in physical activity. Therefore, it is essential to motivate haemodialysis patients to be physically active and adapt their lifestyle, which involves enhancing the appeal of physical exercises and monitoring their activity [4,14]. One innovative solution that can significantly contribute to increasing patient motivation is the combination of physical training with the virtual reality (VR) technology during haemodialysis sessions. VR offers computer-generated interactive experiences based on the visualization of three-dimensional objects, events, and tasks [15,16,17]. The use of VR systems as a complement to rehabilitation therapy allows patients to receive real-time feedback and perform repetitive, functional activities. This is particularly beneficial during haemodialysis sessions [16,17]. Such a combination of physical training with virtual reality during haemodialysis not only effectively utilizes the time spent in dialysis stations but also reduces the monotony of dialysis therapy, motivates for exercise, and provides medical supervision. This brings benefits associated with regular physical activity [15,16,17].

Given the current state of scientific knowledge, there is a clear shortage of comparative studies regarding the relationship between systematic physical activity and IL-6 and sclerostin levels in haemodialysis patient groups. The issue requires further research and a comprehensive approach.

The aim of this study was to comprehensively assess the impact of regular virtual reality-based physical activity on plasma sclerostin and IL-6 levels, as well as on physical performance and the level of physical activity in patients undergoing renal replacement therapy through haemodialysis.

Our study assumed that regular exercise using the NefroVr system would reduce the concentration of SOST and interleukin 6 in plasma.

## 2. Materials and Methods

### 2.1. Patients

One hundred and two (102) patients diagnosed with stage 5 chronic kidney disease receiving hemodialysis as a renal replacement therapy were enrolled in a study conducted from March 2021 to February 2022. Eligibility for participation was determined by a nephrologist, with participants selected and recruited from the Clinic of Nephrology, Transplantology, and Internal Medicine at the Pomeranian Medical University. The study follows a prospective cohort design.

The selection of participants in the research project was based on the qualifying and disqualifying criteria set out in Table 1.

**Figure 1:**
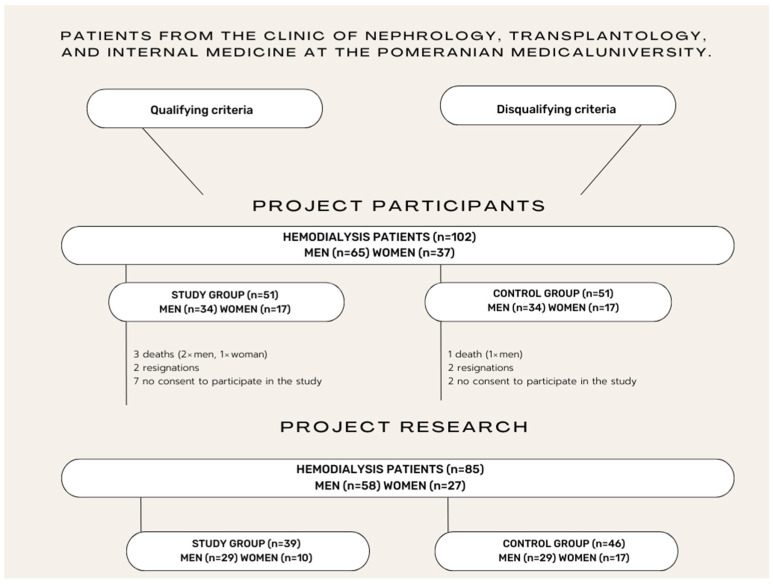
Research qualification scheme.

Ultimately, 85 patients participated in the study. Participants were randomly assigned to a research and control group. A detailed diagram of the process of qualifying patients for the study is presented in Figure 1.

Each patient gave written informed consent to participate in this study. Every effort was made to protect the privacy and anonymity of patients. The study was conducted in accordance with the current version of the Declaration of Helsinki. The study was conducted after obtaining approval from the Bioethics Committee under the number KB-0012/144/2020, issued on 5 October 2020.

### 2.2. Study Process

The patients enrolled in the study were randomly assigned to two groups: the study group and the control group, depending on the type of intervention assigned to them. The study group consisted of patients undergoing haemodialysis as renal replacement therapy, tasked with performing training using the NefroVR system prototype for 20 min during haemodialysis (HD) sessions. Training sessions occurred three times per week, lasting for the first two hours of hemodialysis treatment or until reaching an ultrafiltration (UF) level of 2.5. The control group consisted of patients undergoing hemodialysis as renal replacement therapy without any assigned intervention. The duration of the study period for both the study and control groups was 3 months. 

The NefroVR system prototype, utilized in the research project, consisted of components mounted on a mobile platform with a counterweight. The system comprises several components: a base unit responsible for interconnecting all components and initiating dedicated software, a rehabilitation rotor equipped with a flywheel and adjustable load for exercise during hemodialysis, VR goggles enabling the patient to immerse in virtual reality, a panoramic screen for the patient’s viewing, a touch control screen for the device operator (doctor, nurse, or physiotherapist), and a control set for the patient, which includes a digital joystick and a single button. Functioning through audiovisual stimulation, the device encourages patients to partake in physical activities. Through connectivity to the device, patients engage in a virtual experience, including gameplay, where the sole mode of movement is through the rehabilitation rotor integrated into the device. The rotor’s rotation speed influenced the pace at which the patient participated in the gameplay. It was crucial for the doctor or physiotherapist guiding the patient to have control over adjusting the rotor’s rotations to the game’s speed and the physical resistance of the rotor’s flywheel. These parameters were tailored to the patient’s current health status to ensure the workload was not too intense (e.g., maintaining an acceptable heart rate or avoiding a drop/increase in pressure below/above allowed values). In the clinical tests phase, the patients could choose from a selection of five mini-games, each with a gameplay duration of approximately 20 min. In addition to rotor control, the patient had additional interaction options using a digital joystick and one button. The level of interactivity available to the patient during the clinical trial phase was limited to minimize the time needed to familiarize the patient with working with NefroVR. Fatigue during physical exertion, measured on the Borg scale, was recorded between levels 8–14 for all patients.

At the beginning of the research project (E0) and after 3 months of the study (E3), the level of physical activity and exercise capacity of the patients was assessed. Venous blood was also collected from each patient.

Additionally, at the initiation of the study, patients were required to complete an author-designed survey made for the needs of the study, addressing demographic questions, health status, and lifestyle. Height was measured, from the base to the top of the skull, using a stadiometer attached to an electronic scale. Body weight was measured with a precision of 10 g using the same electronic scale. Weight-to-height ratios were assessed using the Body Mass Index (BMI) [18]. 

All research results were provided to the participants, and each of them had the opportunity to discuss them with appropriate specialists.

#### 2.2.1. Level of Physical Activity

The level of physical activity was assessed on the basis of the International Physical Activity Questionnaire (IPAQ) developed by E. Biernat and R. Stupnicki in 2004 [19]. The questionnaire consisted of seven questions investigating various types of physical activity, considering the place and intensity of the activity. Respondents were informed of a condition necessary for a given activity to be included, namely that each activity considered as physical activity should last for at least 10 min at a time. The respondents considered the last 7 days in the questionnaire. The level of physical activity was calculated and expressed in MET (Metabolic Equivalent of Work). This coefficient was calculated based on the short version of the International Physical Activity Questionnaire. The coefficient was calculated as the sum of intense physical activity, moderate physical activity, and walking-related activity, multiplied by the number of days per week the activity took place, its average duration, and the characteristic coefficient for each activity (Table 2). For intense activity, a constant value of 8.0 was adopted, for moderate activity 4.0, and for walking 3.3 [19].

Based on the survey results, respondents were classified into groups determining their level of physical activity:

(1) High—individuals with 3 or more days of intensive exercise (at least 1500 MET) or 7 or more days of any exercise (at least 3000 MET per week)
(2) Adequate—individuals with 3 or more days of intensive exercise (at least 20 min/day) or 3 or more days of moderate exercise/walking (at least 30 min/day) or 5 or more days of any exercise (at least 600 MET per week)
(3) Insufficient—lack of activity or did not meet the criteria for adequate and high activity [19].

#### 2.2.2. Level of Physical Capacity

Physical capacity was assessed using the 6-min Walk Test (6MWT). The 6MWT was conducted according to the standards of the American Thoracic Society and the European Respiratory Society [20]. The 6MWT measurement was performed along a straight, paved corridor with a length of 30 m, marked at both ends with cones. The distance the patient covered in 6 min was measured, and the results were expressed as an absolute value in meters. 

#### 2.2.3. Blood Sampling

Two (2) millilitres of blood were drawn from the participants. For patients with end-stage renal disease undergoing hemodialysis through the fistula vein, blood samples were collected into EDTA tubes before hemodialysis at the Department of Nephrology, Transplantology, and Internal Medicine of the Medical University of Pomerania during routine blood checks at E0 and E3. The collected blood samples were centrifuged at 4000 rpm for 10 min at a temperature of 4 °C using the MPW—350R centrifuge. Blood plasma was then divided into two separate Eppendorf tubes, with 1.0 mL plasma in each tube (Eppendorf™, Hamburg, Germany), and immediately frozen. Samples were stored at −70 °C until laboratory analysis, using freshly frozen aliquots.

#### 2.2.4. ELISA Tests

Before testing, samples were thawed at room temperature. Initially, plates containing standards were prepared according to the manufacturer’s instructions from Sun Red Biotechnology Company (Shanghai, China). Biotin-labelled antibodies, the test material, and streptavidin were added. The volume of the test material and reagents depended on the analysed parameter. The plates were incubated for 60 min at 37 °C. Next, the plate was washed 5 times with washing buffer. Chromogen A and B was added, and incubated 10 min at 37 °C. Inhibitor solution was added. Absorbance was measured at a wavelength of 450 nm, and the Envision^®^ program was used for analysis based on a linear curve. Plasma concentrations of IL-6 were determined using the commercially available Human interleukin 6 (IL-6) Elisa Kit (Sun Red Biotechnology Company, Shanghai, China). The levels of IL-6 were expressed as [pg/mL]. The sensitivity range for IL-6 was 1 pg/mL, whereas the linearity range was 1.5–300 pg/mL. The plasma sclerostin levels were assessed using the commercially available Human Sclerostin (SOST1) Elisa Kit (Sun Red Biotechnology Company, Shanghai, China). The concentration of sclerostin was expressed in ng/mL, with the sensitivity range of 0.175 ng/mL and the linearity range of 0.2–60 ng/mL.

### 2.3. Statistical Analysis

Statistical analyses were conducted with the use of Statistica 13 (StatSoft, Inc., Tulsa, OK, USA). All data regarding continuous variables are presented as means ± standard deviations (±SD) and medians; qualitative variables are presented as numbers and percentages. Qualitative data were analysed with the Chi-squared test or Chi-squared test with Yates’ correction. The U Mann–Whitney test was applied to compare continuous variables between groups. Laboratory data before and after rehabilitation in the study and control groups were evaluated using the Wilcoxon test. Differences at *p* < 0.05 were considered statistically significant.

## 3. Results

Both the study and control groups comprised patients with end-stage renal disease undergoing hemodialysis as renal replacement therapy, necessitated by the complete absence of diuresis. The age of the examined patients ranged from 35 to 70 years. The ratio of women to men in the study group was 0.34 and in the control group 0.59. Both groups underwent three hemodialysis sessions weekly. The average (standard deviation) duration of a single hemodialysis session was 223.85 (20.47) minutes in the study group and 216.52 (28.92) minutes in the control group.

The demographic data, assessment of concomitant diseases, and habitual behaviour showed no statistically significant differences between the study and control groups, as presented in Table 3.

Table 4 presents an analysis of the differences between the study and control groups. In the evaluation of laboratory data in the first measurement, no statistically significant differences were found between the groups. The second laboratory analysis showed statistically significant differences for IL-6 (*p* = 0.04) and SOST (*p* = 0.03).

The analysis of physical activity of patients from the study and control groups is presented in Table 5. It was shown that patients in the study group had higher physical activity compared to the control group in the second measurement of the conducted study (*p* < 0.001). The analysis of the level of physical activity showed that in the second measurement, the control group’s activity levels varied between each other (*p* = 0.003).

The analysis of the results obtained in the 6-min walk test is presented in Table 6. It was shown that patients in the study group achieved higher scores in the second measurement compared to the control group, and the result was statistically significant (*p* = 0.04). No statistically significant difference was observed between the start and end points of the study in both groups.

## 4. Discussion

In the literature, there are reports of numerous benefits of regular physical activity for the health and well-being of CKD patients [4,15,21,22,23,24]. However, despite this, and despite efforts to make exercises more appealing for this patient group, significantly lower physical activity is still observed compared to individuals with normal kidney function [21,25]. For decades, scientists and clinicians have been conducting research and implementing exercise programs for hemodialysis (HD) patients with the aim of enhancing their health [26].

It is crucial to understand the barriers preventing CKD patients from engaging in regular exercise and identify the most effective strategies to overcome them. The introduction of physical activity monitoring and innovative training methods, as suggested in the literature, seems to be a promising direction. Innovations such as cyclo-ergometer training combined with virtual reality can not only diversify exercises but also help overcome psychological barriers, such as fear of injury or boredom during workouts. The use of modern technologies in exercise programs can also contribute to better progress monitoring and customization of exercises to individual patient needs. Technological innovations, such as combining exercises with VR, may play a crucial role in increasing the attractiveness and effectiveness of exercise programs for this patient group [17,27]. 

The use of the IPAQ in this study provides significant data on the effects of using VR as a tool to support physical activity in haemodialysis patients. The observed increase in physical activity, expressed in MET, after 3 months of such intervention indicates potential benefits of applying modern technologies to promote health in this patient group. It is important to emphasize that, according to the literature review, there are no reports focusing directly on the analysis of physical activity using IPAQ in the context of VR utilization among haemodialysis patients. Studies conducted in haemodialysis patient groups used IPAQ only to determine the relationship between the level of physical activity and the occurrence of sarcopenia [28], cognitive abilities [29], depression [30], cardiovascular disorders [31], and to compare the short version of the IPAQ with accelerometer-based physical activity measurement in haemodialysis patients [32], as well as to assess the plasma levels of 25-hydroxyvitamin D compared to the level of physical activity [33].

The application of the 6MWT in our study allowed the assessment of the functional exercise capacity of patients undergoing haemodialysis. Results indicating higher physical fitness in the study group after exercises using virtual reality are similar to the findings of other studies. They suggest a positive impact of regular exercises on 6MWT outcomes. However, our study adds a new dimension to this research area by focusing on the use of virtual reality as a method supporting the improvement of performance in haemodialysis patients. Rhee et al., as well as Tabibi et al., confirmed an increase in 6MWT performance after aerobic exercises using a cycle-ergometer and resistance exercises [22,34]. Conversely, Arazi et al. demonstrated that breathing exercises applied for 2 months in a group of patients undergoing haemodialysis had no effect on the results of the 6-min walk test [35]. This may suggest that different types of exercises have different effects on endurance of haemodialysis patients, emphasizing the need for individual adaptation of exercise programmes. The outcomes of the study by Kopple et al., indicating that anxiety can lower 6MWT results in the maintenance haemodialysis patient group, are also interesting [36]. The study by Kono et al. focuses on identifying factors influencing 6MWT results, which may be crucial for understanding the aspects affecting exercise tolerance in this patient group [37]. 

The growing number of scientific publications on the impact of physical activity on the levels of pro-inflammatory cytokines, including IL-6, among haemodialysis patients, emphasizes the importance of this research topic [4,38]. The concentration of IL-6 is often elevated in patients with CKD, especially those undergoing dialysis [3,39]. Our study aligns with this research trend, providing valuable information on the impact of physical exercises using virtual reality on the levels of IL-6. At the same time, there is increasing evidence that regular exercise is particularly important for individuals with CKD, as it demonstrates anti-inflammatory effects [38,40]. 

This study revealed a notable difference in IL-6 concentration during the second laboratory test following exercises utilizing virtual reality with the NefroVR system. These findings support the hypothesis that consistent physical activity could lower plasma IL-6 levels. Furthermore, research conducted by Cruz et al. and Liao et al. demonstrated a decrease in IL-6 concentration following regular aerobic exercises [41,42]. Moreover, Meléndez-Oliva et al. demonstrated that regular aerobic and strength exercises can effectively lower IL-6 levels in plasma [43]. These results suggest that regular physical activity, above all, combining different types of exercises, may play a positive role in the modulation of the inflammatory state in haemodialysis patients. 

Mechanical load plays a crucial role in regulating bone formation and remodelling by influencing osteocytes. Mature osteocytes produce and release sclerostin onto the bone surface. Sclerostin acts by inhibiting signals that trigger the proliferation and differentiation of osteoblasts from mesenchymal cells, consequently impeding the formation of new bone [9,44]. There are few publications in the literature regarding the impact of physical training on peripheral blood sclerostin (SKL) levels [9,44,45], and only one study has examined the effect of physical activity on sclerostin levels in a haemodialysis patient group [4]. 

This study has been the first attempt to assess the relationship between regular physical activity in virtual reality using the NefroVR system in a group of patients treated with haemodialysis. It has demonstrated a decrease in SKL concentration after 3 months of exercises. These results are consistent with the study by Turoń-Skrzypińska et al., which showed a negative correlation between SKL concentration and physical activity in haemodialysis patients [4], and with the studies by Ardawi et al., indicating a decrease in SKL concentration along an increase in the duration of physical activity [46]. Conversely, Frings-Meuthen et al. observed an increase in SKL concentration in young healthy men after just 14 days of immobilization in a recumbent position [47].

These findings were confirmed by Gaudio et al. in their study investigating the effect of immobilization on SKL levels in postmenopausal women [48]. Well-planned physical training can not only affect the inflammatory state but also bone metabolism in haemodialysis patients. Given the role of sclerostin as a stimulator of osteoclastogenesis, the observed changes in the concentration of this protein may have clinical significance for the monitoring of and predicting the course of therapy in these patients.

The results of this study emphasize the importance of regular physical activity for patients with CKD undergoing haemodialysis. The use of modern technologies, such as VR and the NefroVR system, has shown improvement in physical activity and functional exercise capacity, as well as a decrease in pro-inflammatory cytokine (IL-6) and sclerostin (SKL). These results indicate potential benefits of implementing innovative exercise methods in this group of patients, which may contribute to improving their health condition and quality of life. At the same time, they highlight the need for further research to fully understand the mechanisms of the impact of physical exercises on various aspects of the health of haemodialysis patients.

### Limitations

The study has had several limitations, including the impact of the COVID-19 pandemic on restricting the study to one dialysis centre and a low number of participants. The health status deterioration of CKD patients, renal replacement therapy, and concomitant diseases also influenced the study outcomes. It was challenging to recruit CKD patients undergoing hemodialysis willing to participate in the project for the evaluation of their physical activity. Additionally, it was challenging to find a suitable physical activity that patients could engage in. The study group’s population decreased over the course of the research project due to factors beyond our control, such as kidney transplantation or the death of participants. Introducing VR may have a psychological impact, potentially encouraging hemodialysis patients to exercise more, which could serve as an independent factor influencing the research process. Moving forward, expanding the size of the study group will be essential to conduct a more comprehensive analysis of the identified correlations.

## 5. Conclusions

Regular physical activity, especially using innovative approaches such as VR, contributes to improving physical fitness and overall activity levels in patients undergoing protracted haemodialysis. The study demonstrated that regular exercise with the application of VR may be associated with a reduction in inflammatory parameters and positive effects on bone metabolism in patients undergoing chronic haemodialysis. Encouraging end-stage renal disease patients undergoing haemodialysis to engage in regular physical exercise may help reduce pro-inflammatory parameters.

## Figures and Tables

**Table 1:** Eligibility and disqualification criteria for participation in the study.

Qualifying Criteria	Disqualifying Criteria
total lack of diuresis, undergoing haemodialysis treatment for at least 3 months (three times a week) age above 18 years.	musculoskeletal disorders preventing participation, severe cardiovascular diseases (heart failure in NYHA class III or IV), acute coronary syndrome within the last three months, uncontrolled hypertension, uncorrectable vision impairments, poorly controlled diabetes (HbA1c above 8% for 3 months), senile dementia, other neurological or psychiatric diseases preventing consent or understanding of the study, nature and participation conditions, malignant tumours, surgeries in the preceding month, lower limb amputation preventing analysis epilepsy

**Table 2:** Formulas for calculating the level of physical activity.

**Physical activity** **in MET min/week**	**=**	**Number of days per week during which physical activity occurred**	**×**	**Average duration of activity**	**×**	**MET value**
**Level of physical activity**	**=**	**MET** **(intensive exercise)**	**+**	**MET** **(moderate exercise)**	**+**	**MET** **(walking)**

**Table 3:** Characteristics of the study and control groups.

		Study Group (n = 39)	Control Group (n = 46)	*p*
Age, mean [SD; Me]		57.56 [17.61; 63.0]	62.63 [15.47; 64.0]	0.27
Sex, n (%)	males	29 (74.36%)	29 (63.04%)	0.26
	females	10 (25.64%)	17 (36.96%)	
BMI (kg/m^2^)		28.23 [5.75; 28.28]	27.89 [5.79; 27.57]	0.76
Professional activity currently, n (%)	no	26 (72.22%)	40 (88.89%)	0.10
	yes	10 (27.78%)	5 (11.11%)	
Professional activity before the start of haemodialysis, n (%)	no	10 (27.78%)	21 (46.67%)	0.08
	yes	26 (72.22%)	24 (53.33%)	
Type of job, n (%)	blue-collar	18 (47.37%)	18 (54.55%)	0.83
	white-collar	11 (28.95%)	7 (21.21%)	
	no job	9 (23.68%)	8 (24.24%)	
Currently smoking cigarettes, n (%)	no	30 (76.92%)	35 (76.09%)	0.87
	yes	9 (23.08%)	11 (23.91%)	
Number of cigarettes per day, mean [SD; Me]		14.44 [6.13; 15.0]	14.09 [7.41; 10.0]	0.68
How many years ago quit smoking, mean [SD; Me]		9.71 [10.95; 5.0]	16.67 [16.17; 13.0]	0.52
Number of HD per week, mean [SD; Me]		2.95 [0.23; 3.0]	2.98 [0.15; 3.0]	0.81
Duration of dialysis [min], mean [SD; Me]		223.85 [20.47; 240.0]	216.52 [28.92; 210.0]	0.11
Concomitant diseases				
Diabetes, n (%)		5 (14.71%)	13 (28.89%)	0.22
Arterial hypertension, n (%)		25 (73.53%)	32 (71.11%)	0.81
Epilepsy, n (%)		4 (12%)	3 (7%)	0.69
Ophthalmic, n (%)		8 (24%)	15 (33%)	0.48
Neurological, n (%)		2 (6%)	3 (7%)	0.75
Treatment with another renal replacement therapy (peritoneal dialysis/kidney transplant) before starting hemodialysis treatment, n (%)		7 (20.59%)	8 (17.78%)	0.98

Legend: BMI—Body Mass Index, HD—haemodialysis, Me—median, n—number of patients, SD—standard deviation, *p*-level of statistical significance.

**Table 4:** Analysis of laboratory data in the study and control groups.

	Study Group (n = 39)			Control Group (n = 46)			*p*
	Mean	Me	SD	Mean	Me	SD	
Laboratory results							
IL-6 Measurement 1	187.83	143.46	146.86	197.53	150.44	127.66	0.35
IL-6 Measurement 2	143.49	131.17	65.09	250.54	159.75	255.98	0.04 *
SOST Measurement 1	17.62	6.84	18.95	16.44	8.38	18.37	0.45
SOST Measurement 2	9.56	7.56	6.53	17.58	9.99	16.15	0.03 *

Legend: Me—median, IL-6—interleukin 6, n—number of patients, SD—standard deviation, *p*-level of statistical significance, * statistical significance.

**Table 5:** Analysis of the physical activity of patients in the study and control groups.

		Study Group (n = 39)	Control Group (n = 46)	*p*
Measurement 1				
Physical activity MET min. week mean [SD; Me]		1734.99 [1616.7; 1254.8]	1354.62 [1457.5; 706.5]	0.16
Level of physical activity MET, n (%)	insufficient	6 (15.79%)	17 (36.96%)	0.08
	adequate	24 (63.16%)	23 (50.00%)	
	high	8 (21.05%)	6 (13.04%)	
Measurement 2				
Physical activity MET min. week mean [SD; Me]		2358.62 [2150.6; 1392.8]	1174.4 [1311.2; 693.0]	<0.001 *
Level of physical activity MET, n (%)	insufficient	3 (8.82%)	19 (42.22%)	0.003 *
	adequate	23 (67.65%)	22 (48.89%)	
	high	8 (23.53%)	4 (8.89%)	

Legend: Me—median, n—number of patients, SD—standard deviation, *p*-level of statistical significance, * statistical significance.

**Table 6:** Analysis of the results of a 6-min walk test in patients in the study and control groups.

	Study Group (n = 39)			Control Group (n = 46)			*p*
	Mean	Me	SD	Mean	Me	SD	
6MWT 1 [m]	406.90	422.50	107.55	372.04	360.00	118.54	0.31
6MWT 2 [m]	432.72	430.00	100.57	364.04	340.00	118.64	0.04 *

Legend: 6MWT—6 Minute Walk Test, Me—median, n—number of patients, SD—standard deviation, *p*-level of statistical significance, * statistical significance.

## Data Availability

The data that support the findings of this study are available from the corresponding author (A.M.), upon reasonable request.
